# Individual and ensemble perception in naturalistic scenes: Effects of context and presentation time

**DOI:** 10.1371/journal.pone.0347430

**Published:** 2026-05-06

**Authors:** Yanina E. Tena Garcia, Bianca R. Baltaretu, Katja Fiehler

**Affiliations:** Experimental Psychology, Justus-Liebig University Giessen, Giessen, Hessen, Germany; Morsani College of Medicine, University of South Florida, UNITED STATES OF AMERICA

## Abstract

In many everyday tasks, we must identify both single objects, as well as object ensembles. Our understanding of the mechanisms behind individual and ensemble perception comes mainly from studies conducted under very simplistic conditions. Here, we aim to further this understanding by moving toward more naturalistic environments. We tested the influence of scene context and presentation time on individual and ensemble perception. Six kitchen objects were presented in two scene contexts, either a kitchen scene or in front of a texturized background for one of three presentation times (100, 800, or 3200 ms). After viewing the objects, participants were instructed to indicate via mouseclick the position of one of the six objects (Individual task) or their average, ensemble position (Ensemble task). We assessed task performance (mouseclicks and eye movements) separately for the two tasks. In the Individual task, objects were located with higher accuracy in the kitchen scene at the longer presentation times. The related eye movements, during initial scene viewing, showed more frequent and larger saccades in the kitchen scene, with no differences in peak velocity, and shorter fixations on individual objects. Increasing presentation time was associated with fewer, larger and slower saccades, as well as longer object fixations. In the Ensemble task, the ensemble position was located more accurately in the texturized background when it was shown briefly (100 ms). Eye movements in the naturalistic scene revealed more frequent, larger and slower saccades, and shorter fixations on the ensemble position. Moreover, increasing presentation time showed fewer, smaller, and slower saccades, with longer fixations on the ensemble region. Overall, we found that scene context and presentation time influence spatial localization and eye movement behavior in individual and ensemble perception, highlighting the need to consider such contextual factors in future work.

## Introduction

One of the key challenges that our visual system faces is extracting task-relevant information within our complex environments. For example, when you wish to make a cup of coffee in your favorite mug, you need to know what it looks like and where it is. This process is referred to individual object perception [1–3]. However, in order to find that single mug in the kitchen, you also need to know where the group of mugs are (i.e., their average position). This is known as ensemble perception [4,5] that provides summary information about a group of objects.

For goal-directed movements toward a specific object, humans rely on individual object perception. An accurate object representation is built up between 100 ms and 2 s [6,7], and depends on several factors, such as object complexity [8], the number of surrounding objects [7,8], and object-context relations [9,10]. Regarding the latter, it has been shown that semantically-congruent scenes (e.g., a hairdryer presented in the bathroom) reduce object processing time [9–11] and facilitate the retrieval of its location [12,13]. This facilitatory effect has been demonstrated for presentation times of four seconds or longer [12,13] and is associated with a general improvement over exposure time [7,14]. Given that our cognitive resources are limited [15–17], scene context and presentation time represent two crucial factors that may facilitate individual object perception [7,9–14]; though, their roles are as yet unknown for object localization under more real-world conditions, especially at shorter presentation times.

To determine summary characteristics about a group of objects, humans mainly rely on ensemble perception. This process relays summary statistics, such as the mean or variance [18], of different features of a group of objects (e.g., average color or identity) [19]. Another feature that has been less studied, but has everyday relevance, is the average location of a group of objects [20–22]. Previous studies that presented abstract stimuli (e.g., dots, lines) showed that their average position can be accurately reported [20,21,23]. Moreover, the average position of several object groups, for example defined by different stimulus colors, can be represented simultaneously [21]. In the real world, being able to determine the location of a group of objects is important in helping you to orient yourself appropriately for a given task. For example, to find the forks in the cutlery drawer in the kitchen, it is crucial to know their average location, in contrast to the position of the spoons or knives. In contrast to individual object perception, the effect of scene context on ensemble perception is less clear. There is some evidence from ensemble perception of facial expressions that a task-irrelevant background (e.g., uniformly oriented lines) that changes between stimulus encoding and information retrieval can reduce ensemble precision [24]. In addition to such contextual factors, presentation time also plays a role. Ensemble perception is known to be a fast and possibly automatic process [4,7,25,26], where object ensembles are built with presentation times as short as 50–500 ms [6,7], depending on stimulus complexity. With longer presentation times (up to 1600 ms), ensemble percepts become more accurate before plateauing, especially for mid- and high-level features like stimulus size and facial features [14,27,28]. However, how effectively these effects transfer to naturalistic scenes is unknown.

In this study, we aimed to close the gap in our understanding of how scene context and presentation time affect individual object and ensemble perception. In a behavioral experiment, participants viewed multiple objects embedded in a kitchen scene (Natural scene) or a texturized background (Non-natural scene) and then, had to indicate either the position of a single object (from a group of six objects; Individual task) or the average, ensemble position (Ensemble task). The objects were presented initially for one of three presentation times (100, 800, or 3200 ms). In the Individual task, we expected better locating performance in the Natural scene [12,13] and at longer presentation times [7,14]. In the Ensemble task, we also expected better locating performance at longer presentation times [14], though we had no a priori hypothesis for scene context (exploratory analysis). Despite limited prior eye-tracking research on these kinds of tasks, eye movements can provide a direct window into how early visual sampling supports later spatial localization [29–31]. Saccade characteristics, like saccade rates, amplitudes and peak velocities, as well as the distribution of gaze across regions of interest (ROIs) reveal how observers allocate attention and extract information during perceptual processing [32–35]. Therefore, we assessed saccade rates, saccade amplitudes, peak velocity and ROI-specific gaze measures for our two perceptual tasks to investigate task-related eye movement behavior and to determine whether scene context and presentation times also play a role early in scene viewing. In brief, we found that, in both the Individual and the Ensemble tasks, locating and eye movement behavior were influenced by scene context and presentation time.

## Materials and methods

### Participants

A group of 76 students from Justus Liebig University (mean age = 23.27 years ± 3.36; 58 females) participated in the experiment. The sample size (N = 76) was determined using a repeated-measures analysis-of-variance (RM-ANOVA) related power analysis (G*Power, $\eta_{p}^{2}=$ 0.035 (f = 0.19), six measurements, α= 0.05, desired power = 0.8). All participants had to meet the following criteria: 1) normal or corrected-to-normal vision, 2) between 18 and 35 years of age, 3) right-handed as indicated by the Edinburgh Handedness Inventory [36] (M = 86.64, SD = 18.07), with 4) no neurological or motor disorders, and 5) intact color vision (verified with Ishihara charts). All participants provided written informed consent and received credit or money (8 euro/h) for their participation. The experiment was conducted in compliance with the guidelines of the local ethical committee at the Department of Psychology, Justus Liebig University Giessen and the Declaration of Helsinki [37]. Data collection started on 23 October 2024 and ended on 13 November 2024.

### Stimuli and scene arrangements

**Fig 1:**
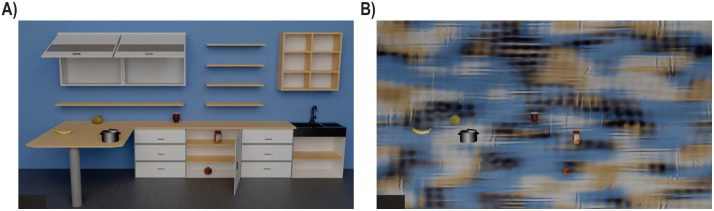
Example stimuli. Example of one object arrangement in the Natural (A) and the Non-natural (B) scenes containing the six target stimuli (left to right: banana, mango, pot, jam jar, pomegranate, and peanut butter jar).

We tested two scene contexts: For the Natural scene, we generated a kitchen scene (created using Blender v2.9; Fig 1A), and for the Non-natural scene, we applied an adapted filter [10] from Portilla and Simoncelli (2000) [38] to the Natural scene to retain low-level information by capturing statistics of brightness, contrast, and patterns across space, orientation, and scale (MATLAB vR2020b; https://de.mathworks.com/products/matlab.html; Fig 1). In each scene context, we presented six kitchen objects. Specifically, we used a banana, mango, pomegranate, jam jar, peanut butter jar, and pot (Blender v2.9; https://www.blender.org/) chosen from a repository (https://www.turbosquid.com/de). We chose to present six target objects, as this is in the upper visual working memory capacity range [15–17] to prevent both floor and ceiling effects in localizing performance. Furthermore, these objects were used to create six object arrangements, where objects were pseudo-randomly placed at physically plausible locations (i.e., on the countertop, on the sink, and/or in the cupboards, open shelves and cabinetry) and in a way that they never occluded one another. In the arrangements, objects assumed different and unique (non-repeating) locations in the scene. We also included six additional arrangements of the same target objects per block, here called ’catch’ scenes, to increase the variability of the arrangements and thus decrease memorization of the six main arrangements. ’Catch’ scenes were not included into the statistical analysis (see Design below for details). Overall, there were 12 different arrangements for each of the two scene contexts, resulting in 24 rendered scenes.

### Apparatus

We ran our paradigm using PsychoPy (v2021.2.0) on an Intel^®^ Core^™^ i5-2500 CPU (3.30 GHz; 8 GB RAM) with NVIDIA^®^ GeForce GTS 450 graphics card, using Windows 10 Pro. Scenes were displayed on a 25” monitor (refresh rate: 60 Hz; resolution: 1920 x 1080 pixels) in a dark room. We ensured a constant distance between the participant and the monitor using a chinrest (distance eye to monitor: 90 cm). For eye movements, we used a video-based desktop mount EyeLink 1000 (SR Research Ltd., Mississauga, Ontario, Canada; sampling rate: 1000 Hz) to record two-dimensional movements of the right eye. The eye-tracker was placed on the table, below the line-of-sight, at a distance of 30 cm to the chinrest. Eye movement accuracy was calibrated and validated before each experimental block with a 5-point grid calibration (threshold for calibration: within 1°; threshold for validation errors: within 0.35°).

### Design

We used a 2 x 2 x 3 within-subject design, with Task (Individual vs. Ensemble), Scene Context (Natural vs. Non-natural), and Presentation Time (100 ms vs. 800 ms vs. 3200 ms) as our three factors. These presentation times were chosen from Neumann et al. (2018), given that this study is most closely matched in terms of stimulus complexity. We blocked Task and Scene Context into four main blocks: 1) Individual, Natural, 2) Individual, Non-natural, 3) Ensemble, Natural, and 4) Ensemble, Non-natural. Each block contained 24 trials: each of the six main scene arrangements was tested at each of the three presentation times, resulting in 18 main trials. Additionally, the six ‘catch’ scenes (see section ’Stimuli and Scene Arrangements’) were also presented once within each block (randomly assigned to the three presentation times). Overall, there were a total of 96 trials distributed across the four blocks. Block order was counterbalanced across participants.

### Procedure

**Fig 2:**
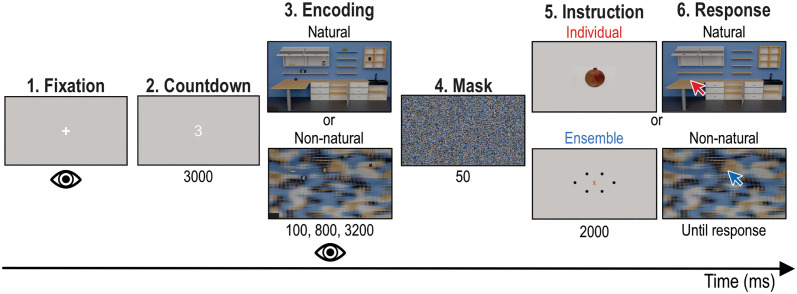
Example trial sequence. In the lower panels, we presented an Ensemble, Non-natural trial, where the location of the ensemble position must be re-produced in the texturized scene. The other two combinations (i.e., Individual, Non-natural and Ensemble, Natural) were also tested. Eye movements were recorded in the Encoding phase and mouseclick endpoints were recorded in the Response phase.

Each of our experimental trials started with an initial Fixation period (Fig 2). Fixation on the cross was required within 3 s of its appearance, otherwise calibration started again. After fixating for 1 s within a 2.5° window, a 3 s Countdown began. Failure to fixate at this stage started the trial anew. The Countdown was followed by the Encoding phase during which a scene arrangement (Natural or Non-natural, depending on the block) was presented at one of the three presentation times (100, 800, or 3200 ms). Then, a 50 ms mask was presented, followed by the 2000 ms Instruction phase that informed about the following Task. In the Individual task, a picture of the target object was presented on the center of the screen. In the Ensemble task, a symbol representing the average position of the objects was shown centrally. In the final, Response phase, the same scene as in the Encoding phase was presented, without any of the target objects. Participants had unlimited time to indicate via mouseclick the respective target position (individual or ensemble). Once they provided their response, a new trial began. We anticipated participants’ preemptive positioning of the mouse from one trial to the next by randomizing the starting position of mouse in one of the four corners of the screen.

### Data processing and analysis

The locating and eye movement data were processed using Jupyter Notebook (v6.2.0; https://jupyter.org/).

#### Locating data.

In order to assess the accuracy of locating responses, we determined the locating error. To do this, we calculated the magnitude of the difference vector between the mouseclick and the object’s actual 2D position. For the Individual task, the 2D position of each object in each scene arrangement was defined relative to its center of mass. For the Ensemble task, we calculated both the center of area (COA; geometric center) and the center of gravity (COG; average location) of the six objects in a scene to determine the ground truth for each scene arrangement [39]. The COA was calculated as the 2D centroid of the polygon formed by connecting the centers of mass of each of the six target objects A, where n represented the six target objects in the scene and *x*_*i*_ and *y*_*i*_ the x and y coordinate of each target, respectively.


$$\begin{array}{cc} A & =\frac{1}{2}\sum_{i=1}^{n}\left(x_{i}y_{i+1}-x_{i+1}y_{i}\right) \end{array}$$ (1.1)

$$\begin{array}{cc} {\text{centroid}}_{X} & =\frac{1}{6A}\sum_{i=1}^{n}\left((x_{i}+x_{i+1})(x_{i}y_{i+1}-x_{i+1}y_{i})\right) \end{array}$$ (1.2)

$$\begin{array}{cc} {\text{centroid}}_{Y} & =\frac{1}{6A}\sum_{i=1}^{n}\left((y_{i}+y_{i+1})(x_{i}y_{i+1}-x_{i+1}y_{i})\right) \end{array}$$ (1.3)

The COG was calculated as the mean X and Y values of all centers of mass of each of the six target objects in the scene.


$$\begin{array}{cc} {\text{mean}}_{X} & =\frac{1}{n}\sum_{i=1}^{n}x_{i} \end{array}$$ (2.1)

$$\begin{array}{cc} {\text{mean}}_{Y} & =\frac{1}{n}\sum_{i=1}^{n}y_{i} \end{array}$$ (2.2)

Participants’ responses were best represented by the COG (S1 Fig and Table in S1 Appendix). Additional comparisons to the screen center were conducted to verify that participants’ responses reflect task-related behavior rather than a general tendency to click near the screen center, as a statistically efficient but imprecise strategy. These comparisons showed that performance was not significantly better described by center-of-screen coding (S1 Fig and Table in S1 Appendix). As such, we used the COG as the ground-truth to calculate participants locating accuracy in the Ensemble tasks. Outlier criteria for the locating analysis were based on both the locating and eye movement data. First, experimental trials were excluded when blinks in the Encoding phase occurred in the 100 ms presentation time (one trial, < 0.01% of the 5472 total trials) or were longer than 200 ms (55 trials, 1% of the total). Second, we observed significant differences in performance in the Individual task for the two red objects (i.e., the jam and pomegranate; S2 Table in S2 Appendix) and therefore excluded the data of these two targets (907 trials, 16.6% of the total). Third, trials were excluded if participants’ response times in the Reproduction phase were shorter than one second or if their locating errors exceeded three standard deviations (calculated separately for each combination of task and presentation time), which led to the exclusion of 82 trials (1.5% of the total). Altogether, we excluded 1045 trials, which resulted in a remaining dataset of 4427 trials (80.9% of the total). We chose to apply linear mixed modeling (LMMs) to our data, given that we wanted to test the factors scene context and presentation times in each task datasets and account for relevant, additional factors, such as repetition number of scene arrangements and participant variability, which could not be fully captured with RM-ANOVAs. LMM analyses were performed separately for our two tasks – one for the Individual and one for the Ensemble task. They were used to test the effects of scene context and presentation time, as well as their interaction, and to capture any potential repetition effects and participant variability. The analysis was conducted using “lmerTest” package and performed in R (v4.2.2; https://www.r-project.org/). Any post-hoc paired t-tests were performed using the “emmeans” package and were Bonferroni-Holm corrected, with p-values written as *p*_BH_. Mean differences (MD), standard errors (SE), t-statistics, p-values and Cohen’s d (d) are provided for each test.

#### Eye movement data.

We examined the effects of scene context and presentation time on eye movement behavior in the two main tasks, specifically during the Encoding phase. While the presentation times were chosen for perceptual effects on the locating behavior, the shortest of the presentation times used here precluded us from assessing any meaningful eye movement behavior [40]. As such, eye movement analysis was restricted to trials that had an 800 or 3200 ms presentation time. First, raw gaze data were low-pass filtered using a second-order Butterworth filter with a cut-off frequency of 30 Hz. Saccade onsets and offsets were identified based on two-dimensional gaze velocity, using a threshold of 30°/s. Only saccades with amplitudes exceeding 0.5°, as defined by the velocity-based onset and offset, and peek velocities below 850 °/s were considered for the analysis. Further analysis was conducted on fixations, which were defined as the time between two consecutive saccades (i.e., the time between the offset of the preceding saccade and the onset of the subsequent saccade). In particular, we included fixations that 1) started after the first saccade after Encoding onset and ended before Encoding offset and 2) were longer than 50 ms and shorter than 2000 ms in the analysis. We excluded data from 8 participants due to technical problems with the eye tracker (interference from eye glasses or contact lenses) and applied our outlier criteria for the eye movement data to the remaining 68 datasets. First, we excluded trials if no saccade was performed during the Encoding phase (4 trials, 0.12% of 3264 total trials) and if blinks in the Encoding phase were longer than 200 ms (55 trials, 1.69% of the total). Second, any trial in which more than 30% of fixations were excluded based on fixation durations shorter than 50 ms or longer than 2000 ms was excluded from further analysis (276 trials, 8.46% of the total). Finally, if more than 50% of trials were excluded based on the first and second criteria, the participant’s data were excluded from the eye movement analysis, resulting in the exclusion of one more participant only for the eye movement analysis. In total, we included data from 67 out of 76 participants in the eye movement analysis (8.52% trials excluded from the remaining 67 datasets).

Using our final data set, we performed analyses on saccade rates, amplitudes and peak velocity as well as on fixation duration on predefined ROIs. For saccade rates, all saccades that met the above listed criteria in a trial were counted and divided by the respective presentation time of the trial. Saccade amplitude was calculated as the straight-line (Euclidean) distance between gaze position at saccade onset and offset,


$$A=\sqrt{(x_{\text{offset}}-x_{\text{onset}}{)}^{2}+(y_{\text{offset}}-y_{\text{onset}}{)}^{2}}\;$$ (3.1)

Peak velocity was defined as the maximum instantaneous velocity within the saccade interval, where instantaneous velocity was computed as the Euclidean norm of the horizontal and vertical velocity components,


$$v(t)=\sqrt{v_{x}(t{)}^{2}+v_{y}(t{)}^{2}},\;v_{\text{peak}}=\max_{t\in [t_{\text{onset}},\;t_{\text{offset}}]}v(t)\;$$ (3.2)

For fixation duration, we determined fixation time spent on predefined ROIs: We defined ROIs for each of the six target objects (individual ROIs) and the ensemble position (ensemble ROI). For the individual ROIs, for each object, a squared space was created based on the extreme boundaries of the object, defined by its uppermost, lowermost, leftmost, and rightmost points. Across stimuli, the average object width was 1.3° of visual angle and the average height was 1.1°. For the ensemble ROI, we created a circular area with a radius of 0.5° of visual angle around the ensemble position and defined this as the ROI. This created a central region with a total diameter of 1° and was done in order to produce an ROI matching that of the individual object ROIs. Fixations (radius of 1°) were considered as landing on the respective ROI if they overlapped with the space of the individual or ensemble ROIs. For saccade rate, amplitude and fixations on ROI we applied the same LMMs like for the locating data (with fixed effects of scene context, presentation time and their interaction, the covariate of scene arrangement repetition and participant variability as random intercepts), separately for the two tasks. For peak velocity we additionally included saccade amplitude as a covariate to statistically control for the amplitude–velocity relationship (the “main sequence”) [41,42].

## Results

### Individual task

First, we looked at the effects of scene context and presentation time on individual object perception. We hypothesized that there would be better locating performance in the Natural compared with the Non-natural scene [12,13] and at longer presentation times [7,14]. For our eye movements, we used exploratory analysis to determine the effects of scene context and presentation time on saccade rates, amplitude and peak velocity as well as on fixation duration on ROIs during Encoding.

#### Locating behavior.

**Fig 3:**
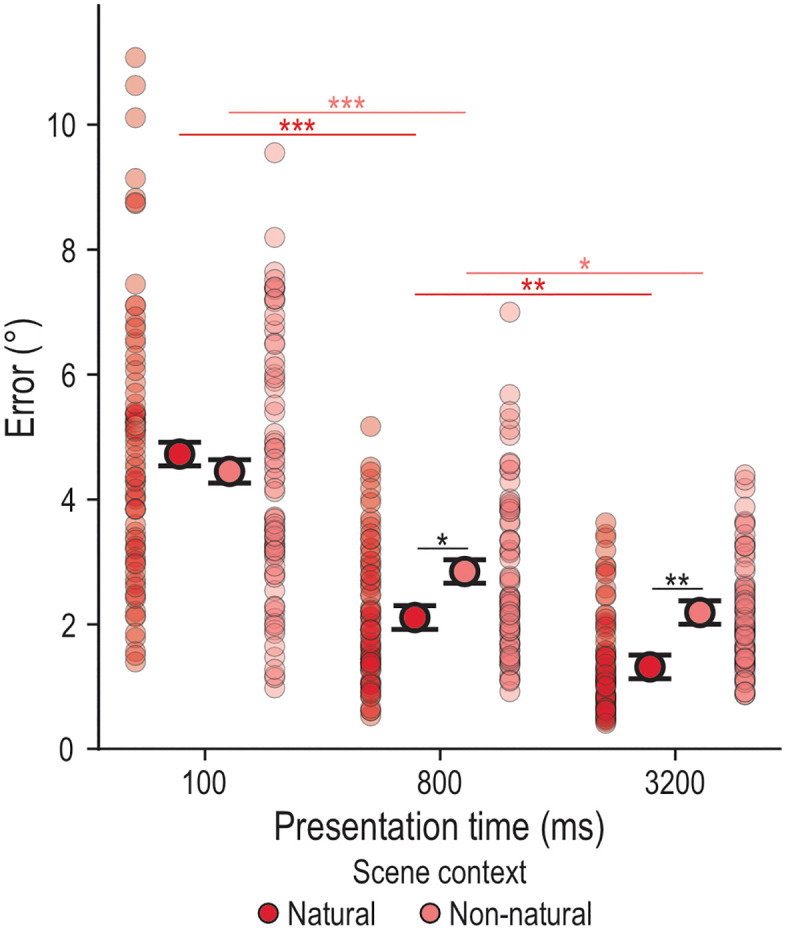
Locating errors in the Individual task. Depicted are the estimated marginal means of the LMM model, represented as black-outlined dots, with the SE as error bars. These are presented alongside the mean locating error per participant per condition, as semi-transparent dots where darker regions indicate greater overlap. Significant differences between conditions are indicated by stars: p < .001 ‘***’, p < .010 ‘**’, p < .050 ‘*’.

We found a significant main effect of scene context (*F*_1,1047.7_ = 8.27, *p* = .004) and of presentation time (*F*_2,1001.1_ = 135.60, *p* < .001), as well as a significant interaction of scene context and presentation time (*F*_2,1001.1_ = 6.15, *p* = .002). We conducted additional post-hoc paired t-tests (Table 1) to test 1) the effect of presentation time within each scene context and 2) the effect of scene context at each presentation time. Across scene contexts, we found better locating behavior from the shortest to the middle presentation times and from the middle to the longest presentation time (Fig 3). Further, when comparing scene context at each presentation time, while we found no difference between the contexts at the shortest presentation time, we did find significant differences at the middle and longer presentation times (i.e., significantly smaller errors in the Natural scene at 800 ms and 3200 ms; Fig 3). We also included a covariate for scene arrangement repetition, which we found to be significant (*F*_11,1169.3_ = 2.45, *p* = .005), showing that locating behavior improved with more repetitions. Overall, these findings indicate that locating individual objects benefits from the Natural scene context with longer presentation time.

**Table 1:** Pairwise Comparisons in the Individual task.

Comparison			MD	SE	*t*	*df*	*p*	*p*_BH_	*d*
100 Nat	–	800 Nat	2.62	0.26	10.24	1001	< .001	< .001	1.09
800 Nat	–	3200 Nat	0.79	0.26	3.09	1130	.002	.008	0.33
100 nNat	–	800 nNat	1.60	0.25	6.38	1000	< .001	< .001	0.67
800 nNat	–	3200 nNat	0.66	0.25	2.60	1000	.009	.018	0.27
100 Nat	–	100 nNat	0.28	0.26	1.08	1021	.282	.282	0.12
800 Nat	–	800 nNat	−0.74	0.26	−2.84	1022	.005	.015	−0.31
3200 Nat	–	3200 nNat	−0.87	0.26	−3.38	1017	.001	.004	−0.36

Scene context (Natural [Nat] and Non-natural [nNat]) post-hoc pairwise t-test results for locating errors across presentation time (800 ms and 3200 ms) in the Individual task. The table reports mean differences (MD), standard error (SE), t-values (t), degrees of freedom (df), uncorrected p-values (p), Bonferroni-Holm adjusted p-values (*p*_BH_), and Cohen’s d (d).

#### Eye movement behavior.

**Fig 4:**
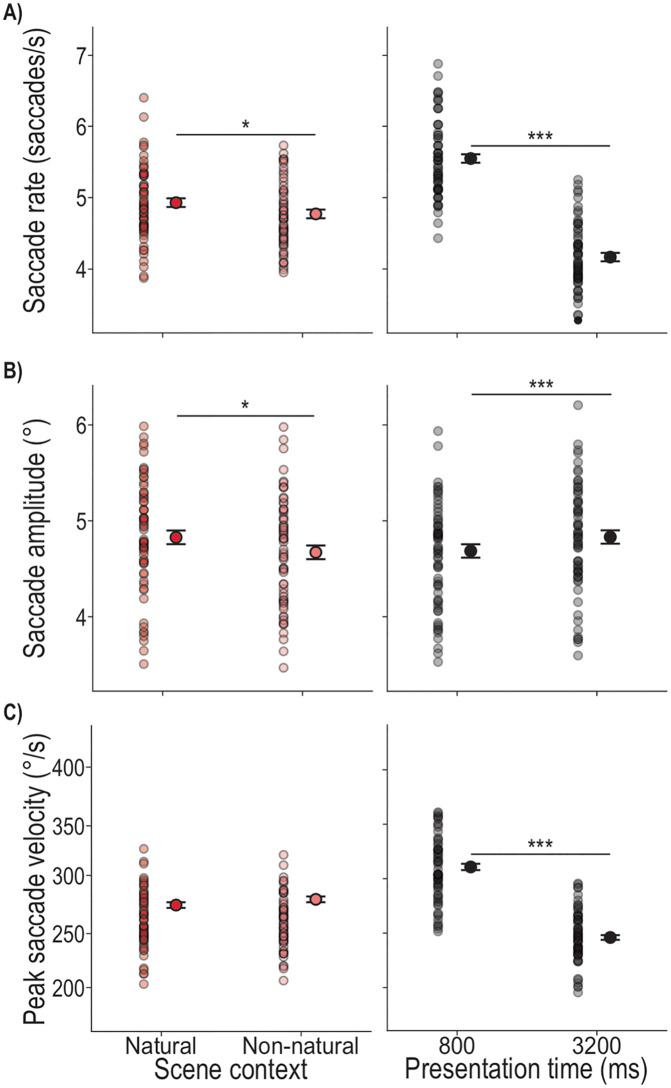
Saccade measures in the Individual task. Depicted are the estimated marginal means of the LMM models, represented as black-outlined dots, with the SE as error bars. These are presented alongside the mean values per participant per condition, as semi-transparent dots where darker regions indicate greater overlap. Significant differences between conditions are indicated by stars: p < .001 ‘***’, p < .010 ‘**’, p < .050 ‘*’.

In our exploratory assessment of eye movements in the Individual task, we investigated whether scene context and presentation time also play a role during Encoding. First, we looked at saccade rates (saccades/s), where we found a significant main effect of scene context (*F*_1,673.7_ = 6.51, *p* = .011), showing higher saccade rates in the Natural (M = 5.19 saccades/s) compared to the Non-natural (M = 5.00 saccades/s) scene context (Fig 4A). We further found a significant main effect of presentation time (*F*_1,635.2_ = 611.53, *p* < .001), showing a higher saccade rate in the 800 ms presentation time conditions (M = 5.93 saccades/s) compared to the 3200 ms conditions (M = 4.23 saccades/s) (Fig 4A). The interaction of scene context and presentation time was not significant (*F*_1,635.5_ = 1.43, *p* = .232) as well as the covariate repetition of scene arrangement (*F*_11,669.0_ = 0.46, *p* = .928). Thus, participants made more saccades during stimulus presentation in the Natural context compared to the Non-natural context, and saccade rates increased at shorter presentation times.

In a next step we examined saccade amplitudes during scene encoding. We found a significant main effect of scene context (*F*_1,667.1_ = 5.86, *p* = .016), with in average 0.16° longer saccade amplitudes in the Natural compared to the Non-natural scene (Fig 4B). There was also a significant main effect of presentation time (*F*_1,634.3_ = 6.27, *p* = .013) showing longer saccade amplitudes in the 3200 ms presentation time condition (M = 4.82°) compared to the 800 ms condition (M = 4.68°) (Fig 4B). The interaction of scene context and presentation time was not significant (*F*_1,635.5_ = 1.43, *p* = .232) as well as the covariate repetition of scene arrangement (*F*_11,669.0_ = 0.46, *p* = .928). Altogether, saccade amplitudes for the Individual task reveal that larger eye movements are made in the Natural compared to the Non-natural scenes and at longer compared to shorter presentation times.

We further analyzed the peak velocity of saccades, while controlling for saccade amplitude. We found a significant main effect of presentation time (*F*_1,13292.2_ = 585.87, *p* < .001) showing faster saccades performed in the 800 ms presentation time condition (M = 310.79°/s) compared to the 3200 ms condition (M = 245.99°/s) (Fig 6C). There was no main effect of scene context (*F*_1,11784.0_ = 3.3, *p* = .070), no significant interaction of scene context and presentation time (*F*_1,13287.7_ = 2.06, *p* = .151) and no significant effect of the covariate repetition of scene arrangement (*F*_11,8263.5_ = 0.59, *p* = .835). We found a significant effect of the covariate saccade amplitude (*F*_1,13287.7_ = 6067.9, *p* < .001), showing larger saccade amplitudes with higher peak velocities. Thus, peak velocity mainly shows an increase at shorter presentation times.

**Fig 5:**
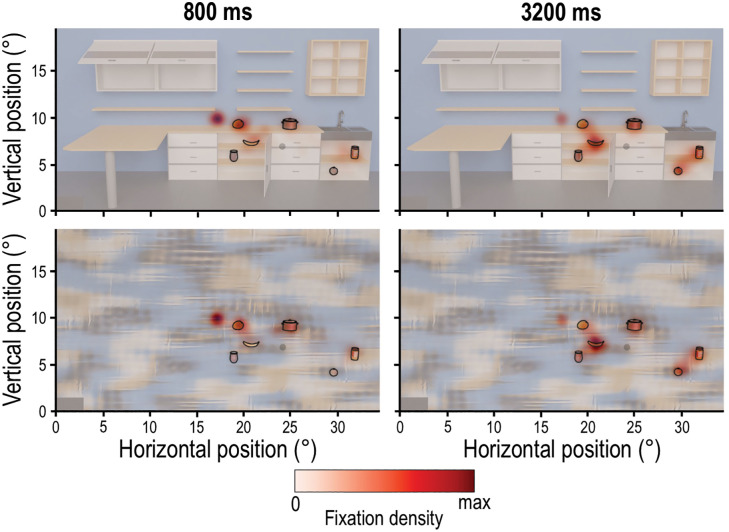
Heatmaps for the Individual task. The heatmaps are separated for scene context (Natural and Non-natural, upper and lower rows, respectively) and for two presentation times (800 ms and 3200 ms, left and right columns, respectively). Darker colors indicate higher fixation density. Colormaps were normalized separately for each condition and thus do not permit direct comparisons of color intensity across heatmaps. Individual target objects are outlined in black.

In addition, we investigated where participants looked during the Encoding phase. To do so, we first used heatmaps to visualize the focus of participants’ gaze for each scene context and the two presentation times (800, 3200 ms). Fig 5 illustrates that, across all four conditions, participants fixated on or near the individual target objects. Moreover, increasing presentation times are accompanied by a spread of fixation behavior, especially toward objects found farther from the center of the screen.

**Fig 6:**
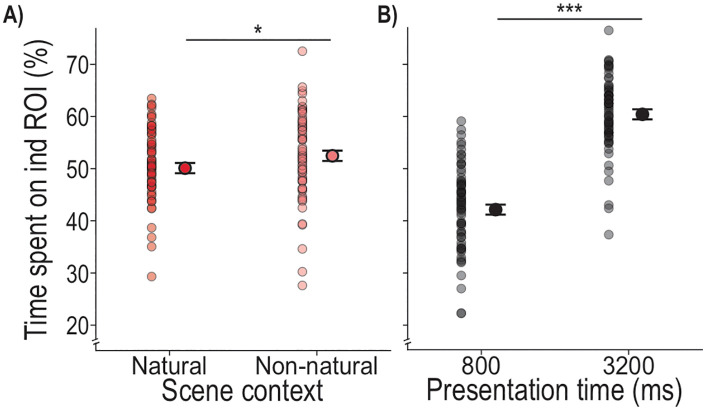
Time spent on individual ROI in the Individual task. Depicted are the estimated marginal means of the LMM model, with the SE as error bars. These are presented alongside the average time participants spent on the ROI per participant per condition, as semi-transparent dots where darker regions indicate greater overlap. Significant differences between conditions are indicated by stars: p < .001 ‘***’, p < .010 ‘**’, p < .050 ‘*’.

Finally, we quantified the spatial distribution of gaze during the Encoding phase by testing the time participants spent fixating any of the six target objects (individual ROIs). We found a significant main effect of scene context (*F*_1,679.8_ = 4.51, *p* = .034), with more fixations on individual objects in the Non-natural scene (M = 52.53%) compared to the Natural scene (M = 50.17%), as can be seen in Fig 6A. We also found a significant main effect of presentation time (*F*_1,636.2_ = 326.86, *p* < .001), with 18.3% more fixations directed toward individual targets in the 3200 ms presentation time (M = 60.48%) condition compared to the 800 ms condition (M = 42.21%) (Fig 6B). There was no interaction of scene context and presentation time (*F*_1,636.5_ = 1.30, *p* = .254), as well as no effect of the covariate repetition of scene arrangements (*F*_11,673.1_ = 0.80, *p* = .645). Thus, in the Individual task, ROI analysis showed that gaze lasts longer on individual target objects in the Non-natural scene context as well as at longer presentation times and may reflect a spread of gaze toward increasingly peripheral targets (Fig 5).

### Ensemble task

For the Ensemble task, we also investigated how scene context and presentation time affect locating behavior. We assumed locating performance to be improved with longer presentation times [7,14], while no a priori assumptions were made regarding the effect of scene context. Given that these two factors have been little studied on eye movement behavior for ensemble perception, we performed the same exploratory analyses as above (see Individual Task – Eye Movement Behavior).

#### Locating behavior.

**Fig 7:**
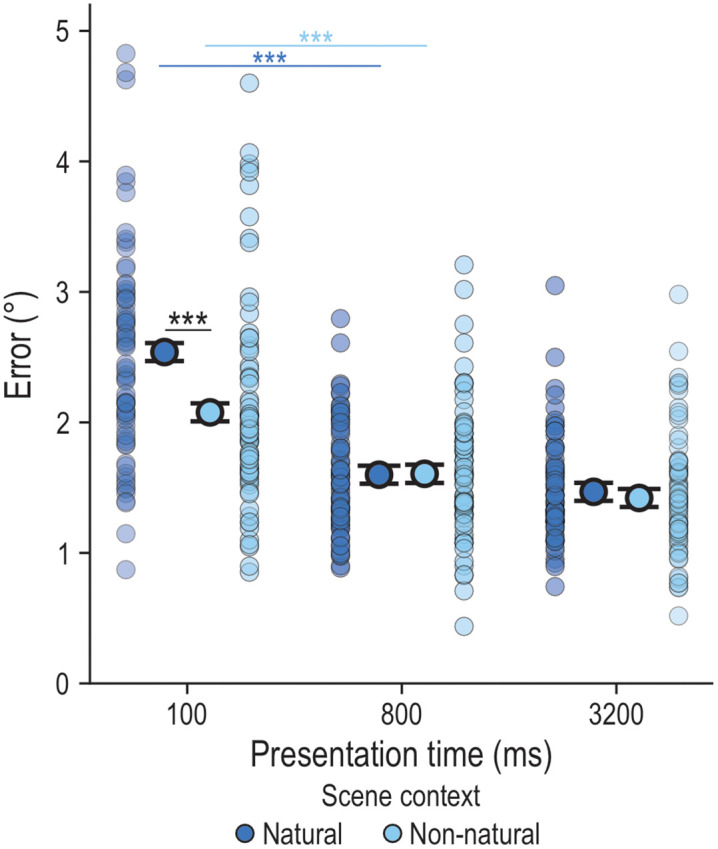
Locating errors in the Ensemble task. Depicted are the estimated marginal means of the LMM model, represented as black-outlined dots, with the SE as error bars. These are presented alongside the mean locating error per participant per condition, as semi-transparent dots where darker regions indicate greater overlap. Significant differences between conditions are indicated by stars: p < .001 ‘***’, p < .010 ‘**’, p < .050 ‘*’.

In testing for the effects of scene context and presentation time on locating performance in the Ensemble task, we found significant main effects of scene context (*F*_1,1137.5_ = 12.06, *p* < .001) and presentation time (*F*_2,1130.1_ = 128.11, *p* < .001), as well as a significant interaction of scene context and presentation time (*F*_2,1130.4_ = 9.98, *p* < .001). We conducted additional post-hoc paired t-tests (Table 2) to test 1) the effect of presentation time within each scene context and 2) the effect of scene context at each presentation time. In both the Natural and Non-natural scene, we found better locating behavior only from the shortest to the middle presentation times (no significant differences between the 800 ms and 3200 ms conditions; Fig 7). This suggests an improvement in locating performance for a limited presentation time range of up to 800 ms that plateaus from 800 across to 3200 ms. We noted a significant difference in scene context, but only at the shortest presentation time, with smaller locating errors in the Non-natural compared to the Natural scene. Just as above, we included in our analyses a covariate of scene arrangement repetition and also found a significant effect (*F*_11,1174.1_ = 4.66, *p* < .001), with locating responses improving across repetitions. Overall, these findings indicate a benefit of the Non-natural scene at short presentation times in locating the ensemble position.

**Table 2:** Pairwise Comparisons in the Ensemble task.

Comparison			MD	SE	*t*	*df*	*p*	*p*_BH_	*d*
100 Nat	–	800 Nat	0.94	0.08	11.54	1130	< .001	< .001	1.15
800 Nat	–	3200 Nat	0.13	0.08	1.62	1130	.106	.318	0.16
100 nNat	–	800 nNat	0.47	0.08	5.78	1130	< .001	< .001	0.67
800 nNat	–	3200 nNat	0.18	0.08	2.26	1131	.024	.096	0.23
100 Nat	–	100 nNat	0.46	0.08	5.66	1133	< .001	< .001	0.56
800 Nat	–	800 nNat	−0.01	0.08	−0.08	1133	.934	.934	−0.01
3200 Nat	–	3200 nNat	0.05	0.08	0.56	1132	.575	1.000	0.06

Scene context (Natural [Nat] and Non-natural [nNat]) post-hoc pairwise t-test results for locating errors across presentation time (800 ms and 3200 ms) in the Individual task. The table reports mean differences (MD), standard error (SE), t-values (t), degrees of freedom (df), uncorrected p-values (p), Bonferroni-Holm adjusted p-values (*p*_BH_), and Cohen’s d (d).

#### Eye movement behavior.

For the analyses of the eye movement behavior in the Ensemble task, we followed a similar analysis pipeline as for the Individual task. We tested for effects of scene context and presentation time on saccade rate, saccade amplitude, peak velocity and fixation durations on the ensemble ROI.

**Fig 8:**
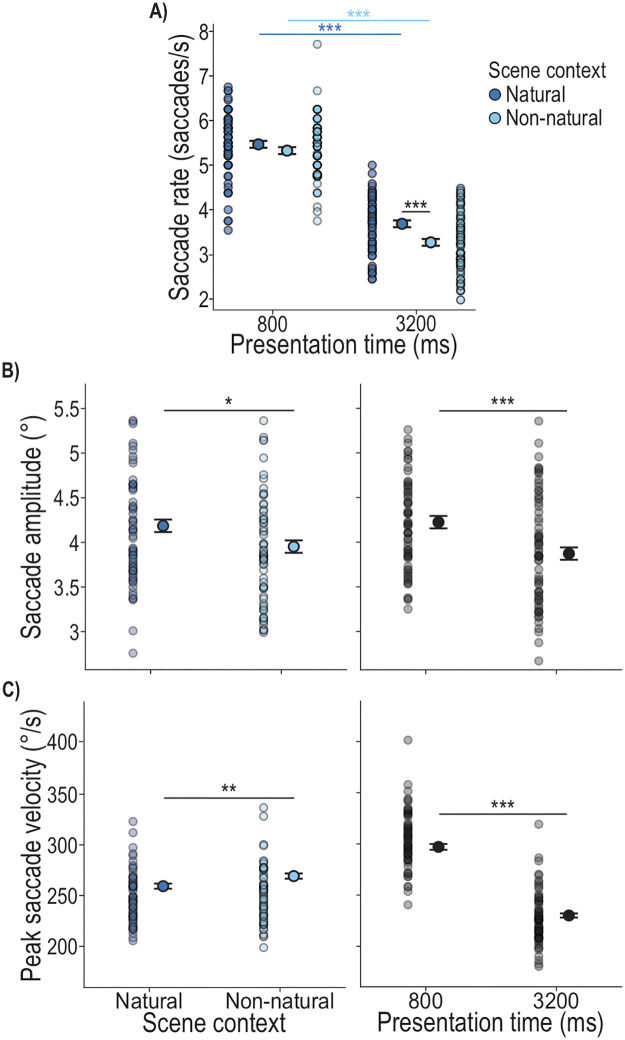
Saccade measures in the Ensemble task. Depicted are the estimated marginal means of the LMM model, represented as black-outlined dots, with the SE as error bar. These are presented alongside the mean values per participant per condition, as semi-transparent dots where darker regions indicate greater overlap. Significant differences between conditions are indicated by stars: p < .001 ‘***’, p < .010 ‘**’, p < .050 ‘*’.

When looking into saccade rates during the Encoding phase, we found significant main effects of scene context (*F*_1,623.1_ = 20.82, *p* < .001) and presentation time (*F*_1,617.7_ = 1087.53, *p* < .001), as well as a significant interaction of the two factors (*F*_1,616.6_ = 5.60, *p* = .018). We analyzed the interaction (Fig 8A) with four post-hoc paired t-tests assessing 1) the effect of scene context at each of the two presentation times (i.e., Natural vs. Non-natural at 800 ms and at 3200 ms) and 2) the effect of presentation time within each scene context (i.e., 800 ms vs. 3200 ms within the Natural context, and within the Non-natural context). We found a higher saccade rate in the Natural compared to Non-natural scene at 3200 ms, but no difference between scenes at 800 ms (800 ms, Natural – Non-natural: MD = 0.14 saccades/s, SE = 0.08, t = 1.66, *p*_BH_ = .344, d = 0.19; 3200 ms, Natural – Non-natural: MD = 0.42 saccades/s, SE = 0.08, t = 4.97, *p*_BH_ < .001, d = 0.54; Fig 8A). In terms of the effect of presentation time on saccade rate, we found a higher saccade rate at shorter presentation times in both scene contexts (Natural, 800 ms – 3200 ms: MD = 1.78 saccades/s, SE = 0.08, t = 21.59, *p*_BH_ < .001, d = 2.34; Non-natural, 800 ms – 3200 ms: MD = 2.06 saccades/s, SE = 0.08, t = 25.10, *p*_BH_ < .001, d = 2.70). The covariate of scene arrangement repetition was not significant (*F*_11,648.7_ = 1.09, *p* = .366). Altogether, saccade rates reveal more saccades in the Natural compared to the Non-natural scenes at longer presentation times (with an overall decrease of saccade rate for longer presentation times).

The analysis of saccade amplitudes during scene encoding in the Ensemble task showed a significant main effect of scene context (*F*_1,621.1_ = 15.33, *p* < .001), with in average 0.23° longer saccade amplitudes in the Natural compared to the Non-natural scene (Fig 8B). There was also a significant main effect of presentation time (*F*_1,616.8_ = 38.68, *p* < .001) showing longer saccade amplitudes for the 800 ms (M = 4.17°) compared to the 3200 ms presentation time (M = 3.93°) (Fig 8B). The interaction of scene context and presentation time was not significant (*F*_1,615.9_ = 0.10, *p* = .757). The covariate repetition of scene arrangement was significant (*F*_11,643.3_ = 1.82, *p* = .047), however, post-hoc comparisons showed no consistent pattern of amplitude decrease or increase across repetitions. Overall, saccade amplitudes reveal that larger eye movements are made in the Natural compared to the Non-natural scenes and at shorter compared to longer presentation times.

For the final analysis of saccadic eye movements we looked into the peak velocity of saccades performed during Encoding. We found a significant main effect of scene context (*F*_1,11232_ = 10.26, *p* = .001) showing on average 9.84°/s faster saccades performed in the Non-natural compared to the Natural scene context (Fig 8C). There was further a significant main effect of presentation time (*F*_1,11287_ = 512.47, *p* < .001) showing faster saccades performed for the 800 ms (M = 297.36°/s) compared to the 3200 ms presentation time (M = 229.90°/s) (Fig 8C). There was no significant interaction of scene context and presentation time (*F*_1,11277_ = 2.57, *p* = .109) and the covariate of repetition of scene arrangement was not significant (*F*_11,5210_ = 1.02, *p* = .034). Similar to peak velocity in the Individual task, the covariate saccade amplitude was significan (*F*_1,10853_ = 4778.20, *p* < .001) showing higher saccade amplitudes with higher peak velocities. Altogether, peak velocity in the Ensemble task revealed that, after controlling for saccade amplitude, saccades were executed quicker in Non-natural compared to Natural scenes and during shorter compared to longer presentation times.

**Fig 9:**
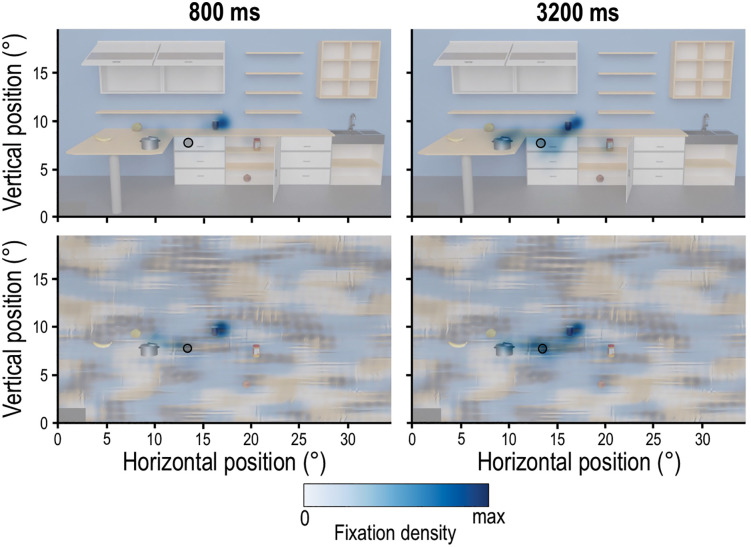
Heatmaps of the Ensemble task. Presented are fixations, averaged across all participants, which are further separated by scene context (Natural and Non-natural, lower and upper rows, respectively) and presentation time (800 and 3200 ms, left and right columns, respectively). The ensemble ROI (average position of all six objects with an additional 0.5° radius) is depicted by the black circle. Darker colors indicate higher fixation density. Colormaps were normalized separately for each condition and thus do not permit direct comparisons of color intensity across heatmaps.

Next to the saccadic eye movements we, also, represented the spatial distribution of fixations during the Encoding phase for the Ensemble task via heatmaps for the scene contexts and presentation times. Fig 9 illustrates that fixations largely fall on individual target objects and that the ensemble position (marked by the black circle) receives more fixations at the longest presentation time across both scene contexts.

**Fig 10:**
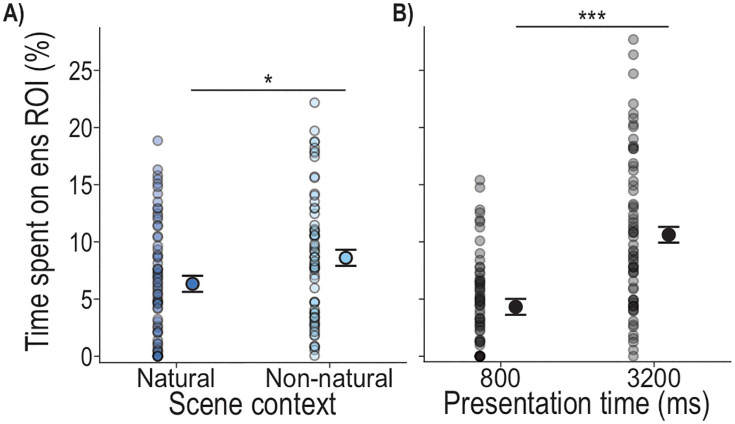
Time spent on ensemble ROI in the Ensemble task. Depicted are the estimated marginal means of the LMM model, represented as black-outlined dots, with the SE as error bars. These are presented alongside the mean time spent on the ROI per participant per condition, as semi-transparent dots where darker regions indicate greater overlap. Significant differences between conditions are indicated by stars: p < .001 ‘***’, p < .010 ‘**’, p < .050 ‘*’.

To quantify how long participants spent looking at the target, we tested for the effects of scene context and presentation time on fixation durations for the ensemble ROI. We found a significant main effect of scene context (*F*_1,631.5_ = 6.51, *p* = .011), with more fixations in the Non-natural (M = 8.61%) compared to the Natural scene (M = 6.33%) (Fig 10A). We also found a significant main effect of presentation time (*F*_1,622.2_ = 54.88, *p* < .001), with 6.3% more fixation time spent on the ensemble position in the 3200 ms condition compared to the 800 ms condition (Fig 10B). Notably, of the entire Encoding phase, participants spent only about 4% looking directly at the ensemble position in the 800 ms condition and about 11% in the 3200 ms condition. We found no additional interaction between scene context and presentation time (*F*_1,620.0_ = 0.27, *p* = .605), and no effect of scene arrangement repetition (*F*_11,657.6_ = 1.08, *p* = .377). In the Ensemble task, fixations on the ensemble ROI increased with more presentation time and occurred more often in the Non-natural compared to the Natural scene context. Notably, fixations on the ensemble ROIs represent only a small proportion of the entire Encoding phase.

## Discussion

In daily life, we constantly locate individual objects and groups of objects, e.g., to grasp a cup or select the shelf with the tea boxes. Here, we tested how individual and ensemble perception are influenced by scene context and presentation time. In the Individual task, we found that locating accuracy was higher in the Natural scene at the mid-long presentation times. Eye movement behavior also showed higher saccade rates and amplitudes in the Natural scene. However, the specific individual ROIs were fixated for longer in the Non-natural scene, and with increasing presentation time. For the longest presentation time, lower saccade rates, larger amplitudes and slower saccades were performed. In the Ensemble task, locating performance improved quickly with increasing presentation time and then, plateaued from 800 ms onward. Interestingly, at the shortest presentation time, locating accuracy was higher in the Non-natural than the Natural scene. In contrast to the plateau effect in the locating behavior, eye movements differed between the mid-long and longest presentation time. In the Natural scene, saccade rates were lower, amplitudes were higher and peak velocity was slower. At the mid-long presentation time, saccade rates and amplitudes were higher and saccades performed quicker. Ensemble ROIs were fixated for longer in the Non-natural scene, and at the longest presentation time. Together, we show that scene context and presentation time are important factors that influence individual object and ensemble perception.

### Individual Task

We found that individual targets were located more accurately when objects were embedded in a naturalistic kitchen scene (Natural scene) and presented for longer periods of time (≥ 800 ms) (Fig 3). This supports previous findings showing that objects embedded in contextually congruent scenes (e.g., hairdryer in bathroom) exhibit a benefit in object recognition and memory-guided localization [9–13]. In particular, the layout of our naturalistic scene may have provided meaningful information (e.g., spatial landmarks) [43,44] during both the Encoding and the Response phases (potentially, offering retrieval cues) that is absent in the texturized background. This may also be related to our lifetime experience, which allows us to build up scene heuristics (i.e., expectations about scene layout, object groupings, and specific object locations) that may reduce the search space and thus, facilitate object localization (particularly, when the object and scene are semantically congruent) [43]. However, we did not observe such a natural scene advantage at our shortest presentation time of 100 ms (Fig 3), probably due to the fact that, at shorter presentation times, there might be insufficient time to successfully encode multiple target objects [7,8,14] or to fully engage scene heuristics. Overall, these results indicate that individual object localization is temporally dynamic and benefits from naturalistic, semantically congruent scene context at longer exposure times.

The results of the locating performance are complemented by the eye movements during the Encoding phase. Here, we found a higher saccade rate performed in the Natural compared to the Non-natural scenes (Fig 4A). This likely reflects the higher complexity of the Natural scene, requiring additional eye movements [45,46], and/or the possibility that the Non-natural texturized background may have facilitated access to task-relevant information, enabling more efficient gaze behavior [46]. Furthermore, at the shorter presentation time of 800 ms, the saccade rate was higher compared to the 3200 ms presentation time (Fig 4A). In combination with the smaller saccade amplitudes and higher peak velocity (Fig 4B and C), this indicates a shift towards faster oculomotor sampling under temporal constraints [47,48]. Taken together, limited viewing time in this experimental paradigm appears to bias participants behavior to prioritizing rapid information acquisition over spatially extensive exploration. In contrast, the larger saccade amplitudes observed for 3200 ms presentation time, together with the heatmap evidence showing increased fixations on more peripheral objects with longer presentation time (Fig 5), suggest that extended viewing time leads to broader spatial exploration. This shift is consistent with a more comprehensive sampling strategy that may support more accurate object localization when temporal constraints are reduced. In addition, we observed more time spent fixating on potential target objects with longer presentation time, increasing from 40% to 60% for presentation times of 800–3200 ms, respectively (Fig 6B) and in the Non-natural compared to the Natural scene (∼ 2%; Fig 6A). Notably, the slightly increased fixation duration on the targets in the Non-natural scene did not confer an advantage for the locating task. Although fixating directly on a target can improve its processing and recall [31], eye movements do not necessarily index task-specific strategies, particularly when task demands allow target information to be gathered peripherally [33,49]. Altogether, our results show scene-dependent differences in eye movements that do not directly relate to task performance.

Overall, the results indicate that individual object perception benefit from naturalistic scenes with longer presentation times. Eye movements were also affected by scene context and presentation time, suggesting increased engagement in more complex, naturalistic scenes.

### Ensemble task

To date, the impact of naturalistic scene context on ensemble perception has not yet been explored. What has been shown thus far is that even simple backgrounds (e.g., oriented lines) can provide contextual information and ultimately influence ensemble perception (e.g., of faces) [24]. Here, we directly compared the influence of simplistic (Non-natural) and naturalistic (Natural) scene context on ensemble perception (Fig 2). In our Ensemble task, we found more accurate locating performance in the Non-natural scene context, particularly at the shortest presentation time (Fig 7). The lack of a Natural scene benefit might be due to the fact that the ensemble position is neither spatially constrained by the kitchen scene nor by expectations derived from the naturalistic context. Further, the fact that this effect was found at the shortest presentation time supports the notion that ensemble perception can operate on short timescales [6,7] (from as short as 100 ms). Moreover, the embedding of the targets within 3D-rendered scenes (i.e., within a kitchen) might have increased the difficulty of object extraction, especially at very short presentation times [50–52]. Longer presentation times (≥ 800 ms) may allow for a more detailed visual analysis that can facilitate ensemble perception (Fig 7) [14]. Future investigations should focus on the temporal evolution of ensemble perception, as a function of the richness of naturalistic scenes.

To date, eye movement behavior in ensemble tasks is widely unexplored. We found that saccade rate was affected by both scene context and presentation time. More saccades in respect to the presentation time were performed in the Natural compared to the Non-natural scene at the longest presentation time (3200 ms) (Fig 8A), which likely reflects greater complexity of the Natural scene [45,46], and/or more efficient gaze behavior enabled by the Non-natural texturized background [46]. This interpretation is further supported by the larger saccade amplitudes in the Natural scene context compared to the Non-natural scene, likely reflecting the need for broader gaze shifts to locate the targets, whereas the unstructured background provides fewer additional (contextual) cues and thus requires less extensive exploration. Consistent with this interpretation, peak velocities were higher in the Non-natural scenes. Natural scenes typically contain meaningful objects and spatial structure that guide gaze toward specific regions of interest and may require more detailed visual inspection. In contrast, the lack of informative structure in the simplistic backgrounds may encourage a more exploratory scanning strategy, allowing gaze shifts to be executed more rapidly. Similar to the Individual task also for the Ensemble task the saccade rate was higher in the 800 ms compared to the 3200 ms presentation time in both scene contexts which possibly indicates the higher time pressure to perceive the important spatial information in the 800 ms condition [47,48]. This is further represented in the overall quicker saccades performed in the 800 ms condition (Fig 8C). The larger saccade amplitudes observed at shorter presentation times as well as the fixation allocations in the scene (Fig 9) suggest that, in the Ensemble task, observers prioritize rapid, large-amplitude eye movements to quickly sample informative scene regions, such as individual objects, early in viewing. In contrast, longer viewing durations reduce the need for extensive exploration, shifting the focus toward the group’s mean position (Fig 9) once the relevant information has been localized. Fixation duration on the ensemble ROI was also influenced by scene context and by presentation time, resulting in more time spent on the ensemble position in the Non-natural scene and at longer presentation time (Fig 10). Longer fixations on the ensemble ROI in the Non-natural scene may reflect less of the need to parse more information to extract the ensemble position [45,49]. However, it didn’t confer an advantage for the locating task. While fixations on the ensemble ROI accounted for only 3% to 10% of the Encoding phase, heatmaps suggest that participants also looked at the individual target objects during this phase (Fig 10). This leaves open questions about ensemble percepts emergence [14,53–55]: Is (initial) individual object localization necessary for the production of the ensemble percept [14,53–55] or might this serve the later stages of perceptual representation via noise reduction, especially in naturalistic scenes? Future investigations may focus on further uncovering how ensemble percepts are formed in spatial localization tasks and how eye movements contribute to this process.

### Experimental approach considerations

Regarding the study design, both the present study and the work by Melcher and colleagues [7] tested individual and ensemble perception in separate blocks. In contrast, other studies used designs in which participants did not know in advance which information they would need to report [14]. Previous work has shown that prior knowledge about the type of information to be extracted can influence task performance [26,56]. Consequently, task structure and expectations must be considered when comparing results across presentation times. For this reason, we did not directly compare performance between the Individual and Ensemble tasks. Importantly, the two tasks also differed in response specificity. In the Ensemble task, participants had to report a single target location, whereas in the Individual task the response reduced the target to one of six possible objects. This difference may introduce confounding variation in task difficulty, further limiting direct comparisons between tasks. Nevertheless, consistent with findings obtained using both blocked and mixed designs, our results show that performance improved with increasing presentation time for both individual and ensemble perception [7,14]. We also acknowledge the possibility of learning effects across the experiment, as the same scenes were presented multiple times. To account for this, the number of stimulus repetitions was included as a covariate in the LMM analyses. Although this covariate reached statistical significance for the locating analyses, the critical effects reported above remained robust when controlling for repetition. This suggests that the main findings cannot be explained solely by learning effects. Given the differences in task demands and the potential influence of design choices such as blocking and stimulus repetition, future work using alternative task structures may allow for a more direct comparison between individual and ensemble perception across conditions.

## Conclusion

Overall, we found that individual and ensemble perception of object locations are subject to effects of scene context and presentation time. Individual object perception improved within naturalistic scenes, especially at longer presentation times. Ensemble perception had better performance within the texturized scene, in particular at the shortest presentation times. Eye movements also revealed that saccade behavior (rate, amplitude, peak velocity) as well as fixation allocation are subject to modulations of scene context and presentation time. Future investigations may shed light on how these processes contribute to different everyday behaviors in our complex environments.

## Supporting information

S1 Appendix Reference point comparison for Ensemble task. Results of post-hoc pairwise t-tests comparing locating error between the three different reference points center-of-gravity (COG), center-of-area (COA) and the screen center (SC).
(PDF)

S2 Appendix Individual object analysis. Results of post-hoc pairwise t-tests comparing locating error between the six different target objects.
(PDF)
