# Supplementary material for: Individual and ensemble perception in naturalistic scenes: Effects of context and presentation time
Source: PLoS One. 2026 May 6;21(5):e0347430. doi: 10.1371/journal.pone.0347430 (PMC13148687; doi:10.1371/journal.pone.0347430)
Supplement: S1 Appendix — Results of post-hoc pairwise t-tests comparing locating error between the three different reference points center-of-gravity (COG), center-of-area (COA) and the screen center (SC). (PDF) [file pone.0347430.s001.pdf]

S1 Appendix A.   Reference point comparison for Ensemble task

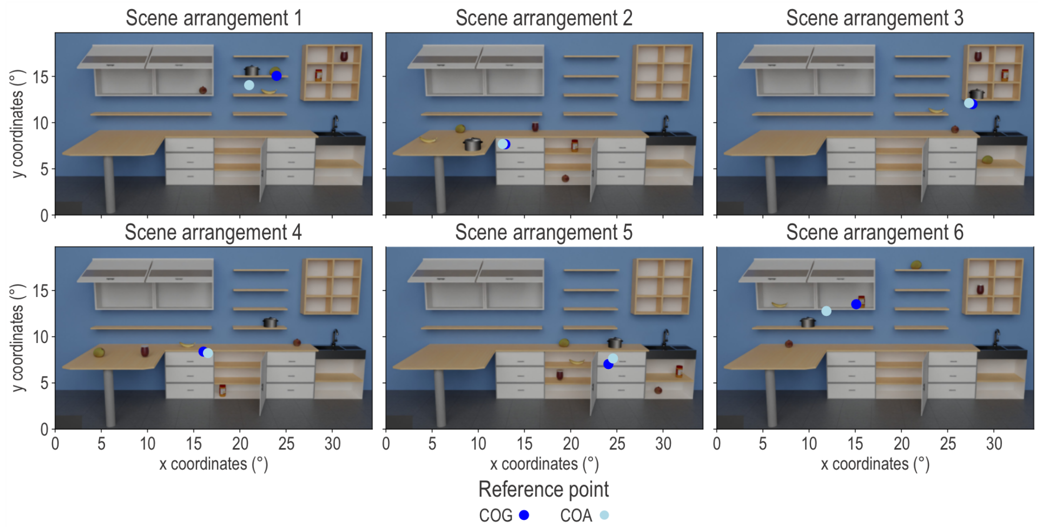

Figure S1: Center of gravity and center of area for each arrangement Depiction of the positions of the center of gravity (COG; dark blue circle) and center of area (COA; light blue circle) for the six scene arrangements used in this study.

The comparison if participants' locating of the ensemble position can be better explained by the center of gravity (COG), the center of area (COA) or the screen center showed a significant main effect of reference point ($F_{2,150} = 3208.81$, $p < .001$), in favor of the COG.

Table S1: Post-hoc pairwise t-tests of reference point comparison

Comparison			MD	t	df	p
COG	–	COA	-0.70	-27.22	75	$< .001$
COG	–	SC	-4.56	-57.72	75	$< .001$
COA	–	SC	-3.87	-58.33	75	$< .001$

Results of post-hoc pairwise t-tests comparing locating error between the three different reference points center-of-gravity (COG), center-of-area (COA) and the screen center (SC). The table reports mean differences, degrees of freedom (df), t-values and uncorrected p-values.
