# Supplementary material for: Individual and ensemble perception in naturalistic scenes: Effects of context and presentation time
Source: PLoS One. 2026 May 6;21(5):e0347430. doi: 10.1371/journal.pone.0347430 (PMC13148687; doi:10.1371/journal.pone.0347430)
Supplement: S2 Appendix — Results of post-hoc pairwise t-tests comparing locating error between the six different target objects. (PDF) [file pone.0347430.s002.pdf]

S2 Appendix B. Individual object analysis

The comparison of the locating performance of the six target objects in the Individual task (banana, jam, mango, peanut butter, pomegranate, pot) showed a significant main effect of target ($F_{5,375} = 12.75$, $p < .001$). Pairwise comparisons showed higher locating errors especially for the objects Jam and Pomegranate, the only two objects that shared a color (here the only two red objects).

Table S2: Post-hoc pairwise t-tests of individual object comparison

Comparison			MD	t	df	p
Jam	–	Banana	2.19	6.54	375	$< .001$
Jam	–	Mango	1.41	4.19	375	$< .001$
Jam	–	Peanutbutter	1.22	3.64	375	.003
Jam	–	Pomegranate	0.39	1.61	375	.246
Jam	–	Pot	1.88	5.60	375	$< .001$
Pomegranate	–	Banana	1.81	5.38	375	$< .001$
Pomegranate	–	Mango	1.02	3.03	375	.003
Pomegranate	–	Peanutbutter	0.83	2.48	375	.014
Pomegranate	–	Pot	1.49	4.44	375	$< .001$
Banana	–	Mango	-0.79	-2.35	375	.019
Banana	–	Peanutbutter	-0.97	-2.90	375	.004
Banana	–	Pot	-0.32	-0.94	375	.349
Mango	–	Peanutbutter	-0.18	-0.55	375	.583
Mango	–	Pot	0.48	1.42	375	.158
Peanutbutter	–	Pot	0.66	1.96	375	.050

Results of post-hoc pairwise t-tests comparing locating error between the six different target objects (Banana, Jam, Mango, Peanut butter, Pomegranate, Pot). The table reports mean differences, degrees of freedom (df), t-values and uncorrected p-values.
